# SARS-CoV-2 Spike Protein S1-Mediated Endothelial Injury and Pro-Inflammatory State Is Amplified by Dihydrotestosterone and Prevented by Mineralocorticoid Antagonism

**DOI:** 10.3390/v13112209

**Published:** 2021-11-03

**Authors:** Nitin Kumar, Yu Zuo, Srilakshmi Yalavarthi, Kristina L. Hunker, Jason S. Knight, Yogendra Kanthi, Andrea T. Obi, Santhi K. Ganesh

**Affiliations:** 1 Department of Internal Medicine, Division of Cardiovascular Medicine, University of Michigan, Ann Arbor, MI 48109, USA; kumarni@med.umich.edu (N.K.); klhunker@med.umich.edu (K.L.H.); yogen.kanthi@nih.gov (Y.K.); 2 Department of Human Genetics, University of Michigan, Ann Arbor, MI 48109, USA; 3 Department of Internal Medicine, Division of Rheumatology, University of Michigan, Ann Arbor, MI 48109, USA; yzu@med.umich.edu (Y.Z.); syalavar@med.umich.edu (S.Y.); jsknight@med.umich.edu (J.S.K.); 4 National Heart, Lung and Blood Institute, Bethesda, MD 20892, USA; 5 Section of Vascular Surgery, Department of Surgery, University of Michigan, Ann Arbor, MI 48109, USA; easta@med.umich.edu

**Keywords:** endothelial injury, androgen, COVID-19, spironolactone, angiotensin receptor blocker, E-selectin

## Abstract

Men are disproportionately affected by the coronavirus disease-2019 (COVID-19), and face higher odds of severe illness and death compared to women. The vascular effects of androgen signaling and inflammatory cytokines in severe acute respiratory syndrome coronavirus-2 (SARS-CoV-2)-mediated endothelial injury are not defined. We determined the effects of SARS-CoV-2 spike protein-mediated endothelial injury under conditions of exposure to androgen dihydrotestosterone (DHT) and tumor necrosis factor-a (TNF-α) and tested potentially therapeutic effects of mineralocorticoid receptor antagonism by spironolactone. Circulating endothelial injury markers VCAM-1 and E-selectin were measured in men and women diagnosed with COVID-19. Exposure of endothelial cells (ECs) in vitro to DHT exacerbated spike protein S1-mediated endothelial injury transcripts for the cell adhesion molecules E-selectin, VCAM-1 and ICAM-1 and anti-fibrinolytic PAI-1 (*p* < 0.05), and increased THP-1 monocyte adhesion to ECs (*p* = 0.032). Spironolactone dramatically reduced DHT+S1-induced endothelial activation. TNF-α exacerbated S1-induced EC activation, which was abrogated by pretreatment with spironolactone. Analysis from patients hospitalized with COVID-19 showed concordant higher circulating VCAM-1 and E-Selectin levels in men, compared to women. A beneficial effect of the FDA-approved drug spironolactone was observed on endothelial cells in vitro, supporting a rationale for further evaluation of mineralocorticoid antagonism as an adjunct treatment in COVID-19.

## 1. Introduction

Severe Acute Respiratory Syndrome Coronavirus-2 (SARS-CoV-2) is a new and rapidly mutating virus causing the COVID-19 pandemic, affecting millions of people globally. SARS-CoV-2 invades human cells by utilizing angiotensin-converting enzyme 2 (ACE2) as a cognate receptor, after being primed by transmembrane protease serine 2 (TMPRSS2), an androgen regulated gene [1,2]. SARS-CoV2 infection is attenuated by anti-ACE2 antibodies, while SARS-CoV infection is enhanced in mice overexpressing ACE2 [3,4]. Likewise, TMPRSS2 knockout mice show reduced SARS-CoV replication and milder lung damage, implying critical roles of ACE2 and TMPRSS2 in SARS coronavirus infection [5,6].

While there is no difference in the proportion of men and women infected with SARS-CoV2, men face higher odds of developing severe illness and death compared to women [7,8,9]. However, the molecular basis of this sex bias in COVID-19 severity has not been precisely defined. Innate and adaptive immune system responses differ in men and women. For instance, the expression of the toll-like receptor 7 (TLR7) gene located on the X chromosome and CD4+/CD8+ T-cell ratio are higher in women, indicating that differences in host immune response may explain a portion of the sex differences in disease severity in COVID-19 [8,10]. Another important X-lined gene in SARS-CoV2 infection is ACE2, which escapes X chromosomal inactivation [10,11], although ACE2 transcript expression is found to be is similar in both men and women in normal tissues [12]. However, in patients with heart failure, a higher circulating ACE2 level was observed in men compared to women, which may be linked to higher shedding of ACE2 from the plasma membrane upon SARS-CoV2 binding [13,14]. Higher circulating plasma concentrations of androgens such as dihydrotestosterone (DHT) and testosterone are present in males as compared to females post-puberty, and evidence suggests that androgens are involved in immune suppression, while estrogen activates immune responses during viral infection [10]. In an observational study, patients with prostate cancer receiving anti-androgen therapy were less likely to be infected with SARS-CoV-2, suggesting a plausible association between androgen levels and COVID-19 disease severity [15]. The global data supporting a consistent sex-bias of COVID-19 illness and death has important implications for the ongoing public health response to this pandemic.

Previous reports have suggested that endothelial activation plays a critical role in COVID-19, promoting systemic and vascular inflammation and subsequent thrombosis [16]. Postmortem histopathological analysis of patients with COVID-19 and multiple organ failure have demonstrated viral inclusion bodies within endothelial cells, with associated vasculitis and endothelial cell death in diverse vascular beds [17,18]. Endothelial activation alters anticoagulant and fibrinolytic homeostasis, and thereby disrupts normal blood fluidity at the blood-vessel interface. Higher circulating levels of soluble VCAM-1 and E-selectin have been reported in patients with COVID-19, and are associated with elevated levels of pro-inflammatory cytokines and chemokines such as tumor necrosis factor-α (TNF-α), indicating endothelial injury [19,20,21,22]. Whether SARS-CoV-2 directly damages the endothelium is not defined, and the role of androgens and TNF-α signaling in SARS-CoV-2-induced endothelial activation have not been previously explored. Spironolactone is used clinically for the treatment of primary hyperaldosteronism and heart failure due to its anti-mineralocorticoid activity, and other beneficial effects are mediated by its off-target effect to inhibit androgen receptor signaling [23]. Early in the COVID-19 pandemic, concerns were raised about the safety of angiotensin receptor blockade in patients with the COVID-19 infection, as it may alter ACE2 expression and therefore enhance virulence, transmission and disease severity [24,25], an issue that later was found to not be clinically apparent [26,27]. Endothelial cell effects of these drugs are important to define, given the evidence for endothelial activation underlying complications of severe COVID-19 infection [16,17].

In this study, we investigated the potential role of androgens and TNF-α signaling in SARS-CoV-2-induced endothelial injury. We employed the functionally active subunit of spike protein S1 of SARS-CoV2 that has shown binding to ACE2 in a cellular model, and this binding was abolished by the co-administration of receptor-binding domain (RBD) or neutralizing antibody to S1 [28]. The spike protein subunit S1 of SARS-CoV2 has previously shown to upregulate intercellular adhesion molecule-1 (ICAM-1) and vascular cell adhesion molecule-1 (VCAM-1) protein expression in human umbilical vein endothelial cells [29], and caused permeability and barrier dysfunction in pulmonary endothelial cells in vitro [30]. Spike protein shedding of SARS-CoV-2 from the surface of the virions and infected cells may play an important role in COVID-19 pathogenesis [31,32] and prior reports support that spike protein alone can elicit some of the disease manifestation in COVID-19 [33,34]. We tested the effects of mineralocorticoid antagonism with spironolactone and angiotensin receptor blockade with valsartan, two FDA approved drugs, to determine effects on endothelial function and inflammation in response to SARS-CoV2 spike-protein S1-induced endothelial injury in vitro. Utilizing molecular and functional assays, we demonstrated that SARS-CoV-2 S1 exacerbated endothelial injury in the presence of DHT and TNF-α in vitro, and this was abrogated by the mineralocorticoid receptor antagonist spironolactone. We validated the top findings from the in vitro studies in human blood samples from patients infected with COVID-19. We observed sex differences in circulating blood levels of the endothelial injury markers VCAM-1 and E-Selectin, which were significantly higher in men as compared to women hospitalized with COVID-19. Our data provide a potential mechanistic link between androgen signaling and COVID-19 disease severity in men, and identify spironolactone as a potential treatment of COVID-19-related vascular damage.

## 2. Materials and Methods

### 2.1. Cell Culture

Pooled primary human aortic endothelial cells (ATCC, Manassas, VA, USA) were cultured in a humidified CO2 incubator at 37 °C in EGM-2 supplemented with 2% FBS, gentamicin and growth factors (Lonza, Basel, Switzerland). Experiments were conducted with cells at passages 5–8. Confluent (70–80%) endothelial monolayers were established in six-well tissue culture plates before serum-starvation in EGM-2 basal media with 0.2% FBS for 6 h. Lyophilized mammalian SARS-CoV2 S1 subunit (Gln14-Arg683, Accession #YP_009724390.1, GeneTex, Irvine, CA, USA) was obtained from 0.22 mm-filtered PBS. Lyophilized S1 protein was reconstituted in distilled water to give a stock concentration of 0.2 mg/mL, as recommended by the manufacturer.

Endothelial cells were treated with full-length recombinant SARS-CoV-2 spike protein S1 for 24 h in serum starvation media before collecting lysate for transcript quantification by qRT-PCR. Quantitative data from three independent technical replicates are expressed in as mean ± s.d in a bar graph.

### 2.2. Androgen Exposure and Spironolactone Therapy on S1-Induced Endothelial Injury 

Endothelial cells were serum-starved and treated as follows: (1) control wells with 0.1% methanol + 0.1% DMSO (0.1% methanol used to dissolve DHT and 0.1% DMSO used to dissolve spironolactone); (2) DHT (500 nM, Sigma-Aldrich, St. Louis, MO, USA, D-073) + 0.1% DMSO; (3) DHT + S1 (25 nM) + 0.1% DMSO; and (4) DHT + S1 + spironolactone (10 mM, Cayman Chemicals, Ann Arbor, MI, USA, #9000324). Working concentrations of DHT, S1 and spironolactone were determined based upon published reports [35,36,37]. Cells were pre-incubated with S1 and spironolactone for 30 min before the addition of DHT. After 24 h of treatment, cells were collected and RNA was extracted for transcript quantification by qRT-PCR of cell adhesion molecules and fibrinolysis proteins.

### 2.3. Monocyte-Endothelium Adhesion Assay

Human aortic endothelial cell monolayers were stimulated in 24-well plates for 24 h with the following conditions before starting the adhesion assay: (1) vehicle control wells with 0.1% methanol + 0.1% DMSO; (2) S1 (25 nM) + 0.1% methanol + 0.1% DMSO; (3) DHT (500 nM) + 0.1% DMSO; (4) DHT + S1 + 0.1% DMSO; and (5) DHT + S1 + spironolactone (10 mM). Human monocyte THP-1 cells (ATCC, VA, USA) labeled with rhod-40AM (AAT Biorequest, Sunnyvale, CA, USA), incubated with an EC monolayer at 1 × 10^6^ THP-1 cells/well and allowed to adhere for 60 min at 37 °C. Non-adherent cells were removed with three washes, and fluorescence intensity was measured. Results were expressed as relative fluorescent units (RFU) and calculated fold-change as compared to the control wells. 

### 2.4. Treatment of TNF-α, Spironolactone and ARB on S1-Induced Endothelial Injury 

The effects of spironolactone and valsartan were tested on S1-induced endothelial injury in the presence of the inflammatory cytokine TNF-α. Following starvation, endothelial cells were exposed to the following treatments: (1) the angiotensin receptor blocker (ARB) valsartan (10 mM, dissolved in DMSO); (2) ARB + TNF-α (5 ng/mL); (3) ARB + TNF-α + S1 (25 nM); and (4) ARB + TNF-α + S1 + spironolactone (10 mM). Working concentrations of TNF-α and valsartan were determined based on previous reports [36,38,39]. Cells were pre-incubated with S1, ARB and spironolactone for 30 min before the addition of TNF-α. In a separate experiment, the effect of S1 was tested in the presence of ARB to evaluate whether S1 causes any further increase in the transcript expression of cell adhesion or anti-fibrinolytic and fibrinolytic markers in primary human aortic endothelial cells. TNF-α and valsartan were purchased from Sigma-Aldrich, St. Louis, MO, USA (Catalog: T0157) and Fisher Scientific, Waltham, MA, USA (Catalog: V01121G), respectively.

### 2.5. qRT-PCR

At the end of the treatment, cells were lysed and total RNA was extracted according to the manufacturer’s instructions (NucleoSpin RNA, Takara Bio USA, Inc., San Jose, CA, USA) that included the treatment with DNase I for 15 min at room temperature to remove genomic DNA contamination. Reverse transcription of 200 ng of total RNA was performed using SuperScript-III first-strand synthesis system (ThermoFisher, Waltham, MA, USA) in a total volume of 20 mL. The exon spanning primers of cell adhesion markers (SELE, VCAM1 and ICAM1) and anti-fibrinolytic and fibrinolytic markers (SERPINE1 and PLAT) were designed (primer 3 software, Supplemental Table S1) and synthesized (Invitrogen, San Diego, CA, USA). cDNA was amplified using PCR master mix with SYBR-Green (Applied Biosystems, Waltham, MA, USA) and data were calculated by the 2 ^−DD^CT method [38] and presented as fold change of transcripts of cell adhesion and anti-fibrinolytic/fibrinolytic markers in human aortic endothelial cells and normalized with the housekeeping RPL37 gene, as compared to control samples.

### 2.6. Human Samples

Peripheral blood samples from patients diagnosed with COVID-19 were collected into either serum separator tubes containing clot activator and serum separator gel or EDTA tubes (plasma) by a trained hospital phlebotomist. Serum and plasma samples were immediately divided into small aliquots and stored at −80 °C until the time of testing. Blood samples were collected at various time during the hospitalization. All patients had a confirmed COVID-19 diagnosis based on U.S. Food and Drug Administration (FDA)-approved RNA testing. The COVID-19 aspects of the study complied with all relevant ethical regulations, and it was approved by the University of Michigan Institutional Review Board (HUM00179409 and HUM00180521).

### 2.7. Measurement of Circulating Endothelial Injury Markers in COVID-19 Patients 

Soluble VCAM-1 in the plasma (EDTA) of COVID-19 patients (n = 11 in cohort 1, Table 1) was quantified by the LEGENDplex™ Multi-analyte flow assay kit (human adhesion molecule panel) according to the manufacturer’s instructions (Biolegend, San Diego, CA, USA). Data acquisition was performed on a Bio-Rad ZE5 Analyzer (Bio-Rad, Hercules, CA, USA). Standard curve and concentrations were calculated with BioLegend’s LEGENDplex™ Data Analysis Software (Biolegend, San Diego, CA, USA). Soluble E-selectin was quantified in the serum of COVID-19 patients (n = 242 in cohort 2, Table 2) using the human E-selectin Duoset ELISA (DY724, R&D systems, Minneapolis, MN, USA) according to the manufacturer’s instructions. Marker results were reported in pg/mL.

### 2.8. Statistical Analysis

All data were reported as mean ± SD unless specified. Normally distributed data were analyzed by two-sided unpaired t-test and skewed data were analyzed by Mann-Whitney test between two groups. Differences between more than two groups were determined by the analysis of variance (ANOVA). Data analysis was performed with GraphPad Prism software version 8 (San Diego, CA, USA). *p* < 0.05 was defined to be statistically significant.

## 3. Results

### 3.1. SARS-CoV-2 Spike Protein S1-Induced Endothelial Injury in Endothelial Cells

**Figure 1:**
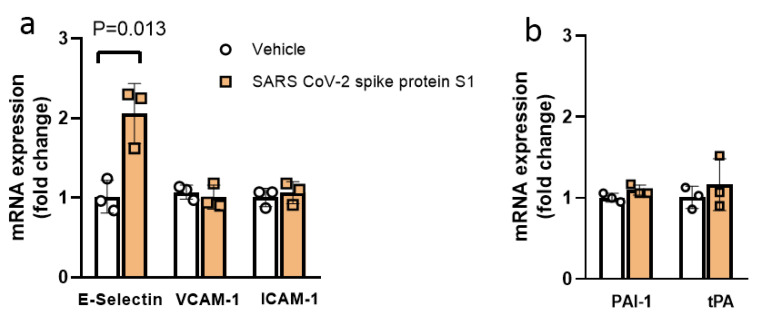
Exposure of SARS-CoV-2 spike protein S1 (S1) increased E-Selectin transcript expression in human aortic endothelial cells (ECs) in vitro. (**a**) S1 (25 nM) exposure alone increased the transcript expression of cell surface adhesion molecule E-Selectin, while VCAM-1 and ICAM-1 were not affected as compared to the vehicle control. (**b**) Anti-fibrinolytic and fibrinolytic gene expression PAI-1 and tPA, respectively were not affected by S1 exposure in ECs in vitro. n = 3 in all groups. Data were analyzed by two-sided unpaired *t*-test. *p* < 0.05 was defined to be statistically significant. All data are expressed as mean ± s.d.

We investigated the effect of exposure to the S1 subunit of SARS-CoV-2 (S1) on endothelial injury in vitro by screening quantitative transcript expression levels of cell surface adhesion proteins [E-selectin, vascular cell adhesion molecule-1 (VCAM-1) and intercellular adhesion molecule-1 (ICAM-1)] and anti-fibrinolytic/fibrinolytic markers [plasminogen activator inhibitor-1(PAI-1)/tissue plasminogen activator (tPA)] in human aortic endothelial cells (ECs) in vitro. S1 exposure increased mRNA transcript expression of E-selectin 2.0-fold, as compared to vehicle (*p* = 0.013, Figure 1a). The transcript expression of VCAM-1 and ICAM-1 were not changed. The transcript expression of anti-fibrinolytic and fibrinolytic markers PAI-1 and tPA, respectively, were also not altered by S1 exposure (Figure 1b).

### 3.2. Dihydrotestosterone (DHT) Exacerbated SARS-CoV-2 S1-Mediated EC Injury and This Effect Was Abrogated by Spironolactone

**Figure 2:**
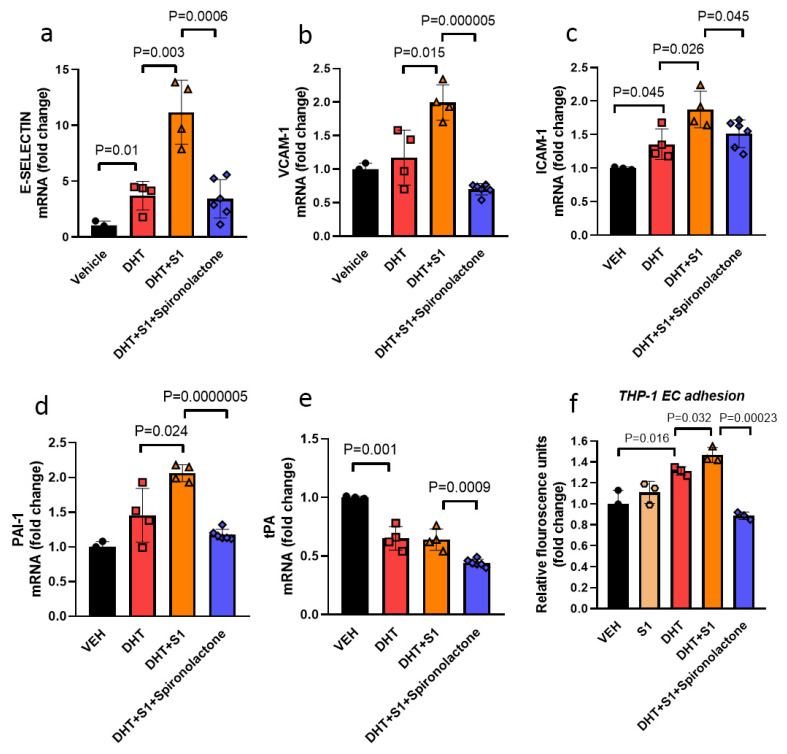
Co-treatment of ECs with SARS-CoV2 S1 with dihydrotestosterone (DHT) in vitro enhanced gene expression of cell adhesion molecules and anti-fibrinolytic marker and promoted leukocyte adhesion, with beneficial effects of spironolactone treatment. (**a**) Transcript expression of cell adhesion molecule E-selectin was increased by DHT alone (500 nM) as compared to the vehicle and expression of E-selectin was enhanced by S1 (25 nM) in the presence of DHT (n = 4). (**b**,**c**) VCAM-1 and ICAM-1 transcript expression were also enhanced by S1 in the presence of DHT as compared to DHT alone. (**d**) Co-treatment of S1 with DHT enhanced PAI-1 transcript expression. (**e**) Transcript expression of fibrinolytic tPA was significantly downregulated by DHT alone, while co-treatment with S1 did not further change tPA transcript expression. (**f**) DHT treatment increased the adhesion of human THP-1 monocytes to EC monolayers, which was further enhanced by the S1 exposure. Spironolactone (10 mM) reduced the transcript expression of cell adhesion molecules (E-selectin, VCAM-1 and ICAM-1) and anti-fibrinolytic/fibrinolytic PAI-1/tPA, respectively, and blocked the adhesion of THP-1 to ECs monolayer in vitro (n = 3–4). ● (Vehicle), ■ (DHT), ▲ (DHT+S1), ♦ (DHT+S1+Spironolactone), ⬣ (S1). Data were analyzed by the analysis of variance (ANOVA). *p* < 0.05 was defined to be statistically significant. All data are expressed as mean ± s.d.

**Figure 3:**
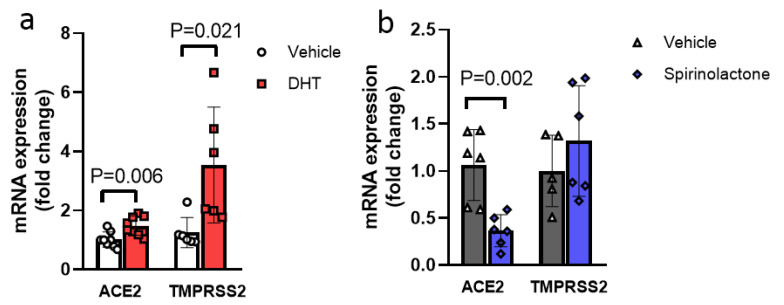
Effects of DHT and spironolactone on endothelial ACE2 and TMPRSS2 transcript expression in ECs. (**a**) DHT (500 nM) significantly increased ACE2 and TMPRSS2 transcript expression as compared to its vehicle. (**b**) Spironolactone (10 mM) treatment significantly reduced ACE2 transcript expression as compared to its control vehicle without affecting TMPRSS2 transcript expression in ECs in vitro (n = 6–8). Data were analyzed by two-sided unpaired *t*-test. *p* < 0.05 was defined to be statistically significant. All data are expressed as mean ± s.d.

Testosterone is endogenously converted in part to estrogen by aromatase and to dihydrotestosterone (DHT) by 5α-reductase. DHT is a potent androgen, and cannot be aromatized to estrogen, thus avoiding confounding estrogenic effects. [39]. DHT treatment alone resulted in increased transcript expression of the endothelial cell adhesion molecules E-selectin and ICAM-1 in ECs and significantly decreased tPA, while PAI-1 transcript expression was not affected by DHT exposure (Figure 2a–e). DHT treatment also led to a 1.5-fold increase in ACE2 (*p* = 0.006) and 2.8-fold increase in TMPRSS2 (*p* = 0.021) transcript expression (Figure 3a). In the presence of DHT, S1 exposure increased the transcript expression of E-selectin 3.0-fold (*p* = 0.03), VCAM-1 1.7-fold (*p* = 0.015) and ICAM-1 1.4-fold (*p* = 0.026) and anti-fibrinolytic PAI-1 1.4-fold (*p* = 0.024), while the tPA level was not affected by the co-treatment of S1 with DHT (Figure 2a–e). The effect of S1 alone is presented in Figure 1, to make the list of experimental conditions less condensed for Figure 2, and to focus on S1 treatment alone on endothelial cells in Figure 1.

We next performed a monocyte-endothelium adhesion assay as a functional follow-up to our mRNA expression findings. Although endothelial exposure to S1 alone did not alter monocyte adhesion compared with controls (Figure 2f), DHT treatment increased adhesion of THP-1 to the endothelial cells, which was further enhanced in the presence of S1 (*p* = 0.032) (Figure 2f).

Spironolactone has been used as an anti-inflammatory and anti-fibrotic treatment in the setting of heart failure and has anti-androgen properties as well [40]. We tested whether spironolactone may alleviate EC injury caused by DHT and S1 in vitro. Treatment of DHT+S1 exposed endothelial cells with spironolactone abrogated EC activation, as evidenced by the dramatic reduction and normalization of adhesion and fibrinolytic transcripts expression, with 3.3-fold reduction in E-Selectin expression as compared to the corresponding exposure without spironolactone (*p* = 0.006). Similarly, there was a 1.7-fold reduction of THP-1 adhesion to the endothelial monolayer after spironolactone treatment (*p* = 0.00023, Figure 2a–f). Spironolactone treatment itself significantly reduced ACE2 transcript expression in ECs (Figure 3b).

### 3.3. ARB Exposure Did Not Change SARS-CoV-2 S1-Mediated EC Injury

**Figure 4:**
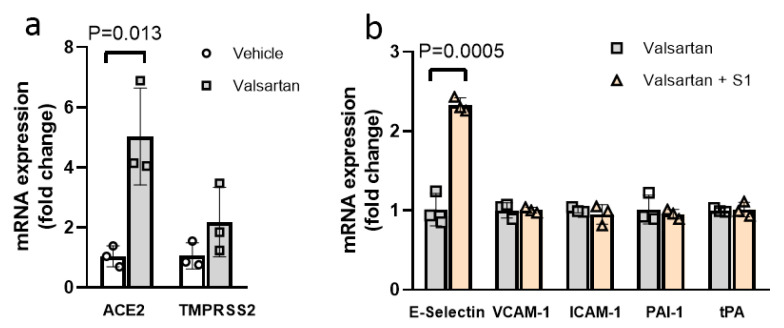
Angiotensin receptor blockade did not exacerbate S1-mediated EC injury in ECs. (**a**) Angiotensin receptor blockade with valsartan (10 mM) significantly increased in vitro EC ACE2 transcript expression without affecting TMPRSS2 expression as compared to the vehicle control. (**b**) In the presence of valsartan, S1 (25 nM) significantly increased the transcript expression of cell surface adhesion molecule E-Selectin, while VCAM-1 and ICAM-1 were not affected. In vitro EC transcript expression of the anti-fibrinolytic and fibrinolytic genes PAI-1 and tPA, respectively, were not affected by S1 exposure in the presence of valsartan. n = 3 in all groups. Data were analyzed by two-sided unpaired *t*-test. *p* < 0.05 was defined to be statistically significant. All data are expressed as mean ± s.d.

Basal transcript expression of ACE2 in ECs was low (Ct~33 ± 3, data not shown), consistent with previous reports [41]. As expected, ARB exposure significantly increased the ACE2 transcript expression in ECs (4.83-fold increase, *p* = 0.013, Figure 4a), though it did not exacerbate S1-mediated EC injury according to transcript expression of endothelial inflammatory markers E-selectin, VCAM-1 and ICAM-1, or anti-fibrinolytic marker PAI-1 and fibrinolytic tPA (Figure 4b). TMPRSS2 transcript expression was similarly low in ECs (Ct ~ 34 ± 3, data not shown), and its expression was not affected by ARB treatment (Figure 4a).

### 3.4. Spike Protein S1 Enhanced TNF-α Mediated EC Injury and Spironolactone Treatment Prevented EC Injury

**Figure 5:**
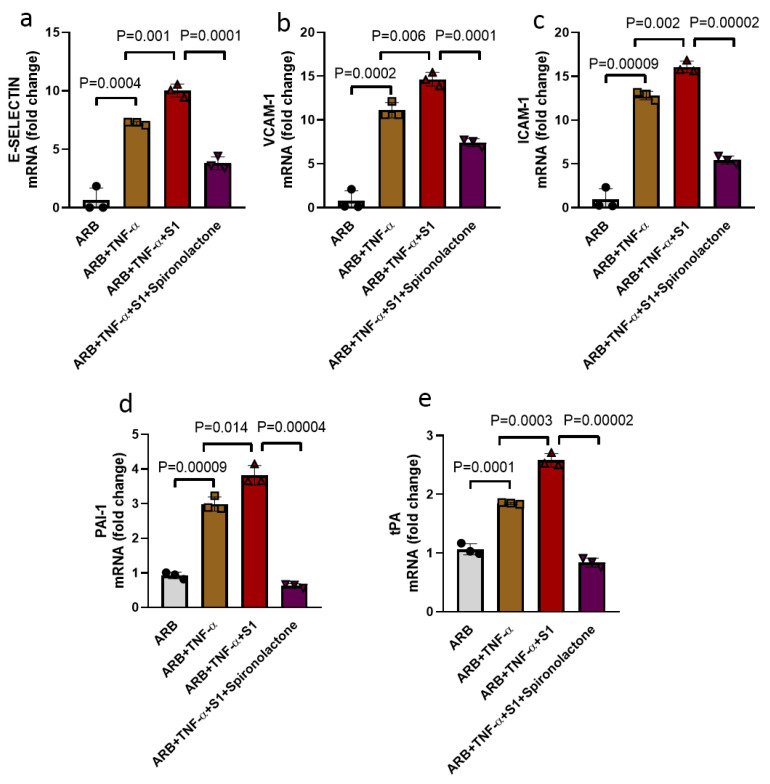
Co-treatment of ECs with S1 and TNF-α enhanced transcript expression of cell adhesion and anti-fibrinolytic/fibrinolytic genes, while spironolactone treatment blocked these effects. (**a**) Transcript expression of cell adhesion molecule E-selectin was increased by TNF-α alone (5 ng/mL) as compared to its control ARB (angiotensin receptor blocker, valsartan) and expression of E-selectin was further enhanced by S1 (25 nM) in the presence of TNF-α. (**b**,**c**) VCAM-1 and ICAM-1 transcript expression were also significantly enhanced by co-treatment of spike protein + TNF-α, as compared to TNF-α. (**d**,**e**) Transcript expression of anti-fibrinolytic and fibrinolytic genes PAI-1 and tPA, respectively, were upregulated by TNF-α, and their expression were further increased by S1 in the presence of TNF-α. Spironolactone (10 mM) markedly reduced both cell adhesion (E-selectin, VCAM-1 and ICAM-1) and anti-fibrinolytic and fibrinolytic PAI-1/tPA genes, respectively, in ECs in vitro, n = 3 in all groups. ● (ARB), ■ (ARB+TNF-α), ▲ (ARB+TNF-α+S1), ▼ (ARB+TNF-α+S1+Spironolactone). Data were analyzed by the analysis of variance (ANOVA). *p* < 0.05 was defined to be statistically significant. All data are expressed as mean ± s.d.

TNF-α has been widely used in vitro studies to elicit pro-inflammatory conditions as observed in hypertension and cardiovascular diseases [42], and as such, TNF-α co-treatment of endothelial cells, along with S1, was used to mimic pre-existing cardiovascular comorbidity in the setting of COVID-19. We tested the effect of TNF-α exposure in S1-mediated EC injury in vitro. Co-treatment of S1 with TNF-α in the presence of valsartan significantly increased the transcript expression of cell adhesion molecule E-selectin (1.38-fold increase, *p* = 0.001), VCAM-1 (1.31-fold increase, *p* = 0.006) and ICAM-1 (1.25-fold increase, *p* = 0.002), as well as anti-fibrinolytic PAI-1 (1.27-fold increase, *p* = 0.014) and fibrinolytic tPA (1.39-fold increase, *p* = 0.0003), compared to TNF-α treatment alone (Figure 5a–f). Expression of VWF, an important common biomarker of endothelial injury, was however not affected by the S1 exposure in the presence of ARB (Figure S1).

We then tested whether spironolactone could provide a similar degree of protection in S1-induced EC injury in the presence of TNF-α, as we observed in androgen treatment. Spironolactone markedly reduced the TNF-α + S1-induced increase in the transcript expression of adhesion protein E-selectin (1.97-fold decrease, *p* = 0.0001), VCAM-1 (2.64-fold decrease, *p* = 0.0001) and ICAM-1 (2.95-fold decrease, *p* = 0.00002), as well as PAI-1 (6.09-fold decrease, *p* = 0.00004) and tPA (3.07-fold decrease, *p* = 0.00002), compared to TNF-α + S1 treatment (Figure 5a–f).

### 3.5. Sex Differences in Endothelial Injury Markers in Men and Women with COVID-19 

**Figure 6:**
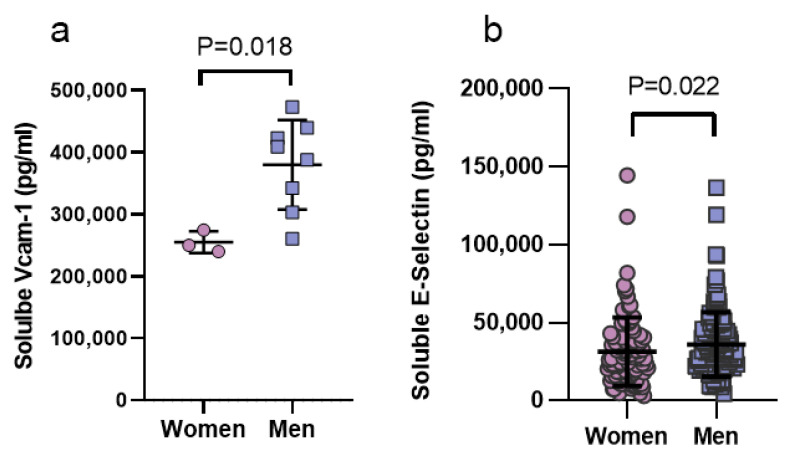
Sex differences in the circulating endothelial injury markers in patients with COVID-19. (**a**) Circulating VCAM-1 protein was significantly elevated in men (n = 7) as compared to women (n = 3) affected by COVID-19. (**b**) Men (n = 136) displayed higher circulating E-Selectin as compared to women (n = 106) in COVID-19. Data were analyzed by Mann-Whitney test between two groups. *p* < 0.05 was defined to be statistically significant. All data are expressed as mean ± s.d.

In order to validate the significance and direction of the in vitro findings, patient-derived samples were accessed to analyze VCAM-1 and E-selectin. Clinical characteristics of patients with COVID-19 infection in the two cohorts analyzed are shown in Table 1 and Table 2, for Cohort #1 and Cohort #2, respectively. We analyzed the protein expression of circulating markers that reflect the endothelial activation (VCAM-1 and E-selectin) in men and women hospitalized with COVID-19. Soluble VCAM-1 was 1.49-fold higher in men as compared to women with COVID-19 (*p* = 0.018, cohort 1, Figure 6a). Sex differences in E-selectin levels did not meet statistical power in Cohort #1, due to low sample size (n = 2 in women, data not shown). Thus, E-selectin levels were measured in an independent cohort, Cohort #2, comprising 242 patients with COVID-19 illness (n = 136 men and 106 women). Levels of soluble E-Selectin were increased in men as compared to women in COVID-19 (1.15-fold increase, *p* = 0.022, Cohort #2, Figure 6b).

## 4. Discussion

The results of the current study support mechanisms of inflammatory activation of the vascular endothelium in COVID-19 infection. Endothelial injury was induced by the SARS-CoV-2 S1 spike protein in vitro, and this effect was exacerbated in the presence of the androgen DHT, that is notably expressed at higher levels in men. Additionally, concurrent exposure to the inflammatory cytokine TNF-α amplified S1-indcued endothelial injury. The in vitro effects were validated first by functional study of leukocyte adhesion to the cultured ECs, which was shown to be increased in the setting of S1 exposure of the ECs in the presence of DHT. Further validation in human samples from patients hospitalized with COVID-19 infection showed that circulating endothelial injury markers VCAM-1 and E-Selectin were concordantly impacted with higher expression in men diagnosed with COVID-19, as compared to women. We found that ARB treatment of ECs in vitro showed no detrimental or therapeutic benefit. Finally, a beneficial effect of mineralocorticoid receptor antagonism by spironolactone was demonstrated, in that it was able to abrogate the worsening endothelial injury by S1 in the presence of DHT or TNF-α in vitro. Taken together, these data qualify endothelial injury in COVID-19, most notably in cellular adhesion molecule expression, contributing to a pro-inflammatory state, and providing vascular correlation to the clinical observation of poorer prognosis and higher mortality in specific patient subgroups, men and individuals with pro-inflammatory conditions such as chronic cardiovascular disease.

Emerging evidence suggests that male sex is a risk factor for COVID-19-related disease severity and mortality, while female sex is somewhat protective, supporting a hypothesis of sex hormone regulation of immune responses during COVID-19 infection [7,8]. A retrospective study of estradiol therapy in postmenopausal women documented a robust improvement in COVID-19-related mortality [43]. In contrast, testosterone may have suppressive effects on the immune system, as studies have demonstrated that androgen deficiency is associated with low regulatory T cells and increased inflammatory cytokines, cytotoxic CD8+ T cells and natural killer cells [10]. Indeed, males have been shown to have a higher viral load in human immunodeficiency virus and hepatitis infection compared to females, suggesting that potential susceptibility in males that may be attributed to hormonal status, although the mechanisms of these findings have not been fully defined [10]. Elevated levels of endothelial cell adhesion molecules promote excessive tissue infiltration of circulating leukocytes, and are associated with inflammation and thrombosis, early critical events reported in COVID-19 infection, which occur at a higher frequency in males [16,44]. Testosterone is the primary androgen in men, which can be partially converted into a more potent form dihydrotestosterone (DHT), as well as estrogen [45]. In a meta-analysis of randomized controlled trials, acute testosterone therapy in hypogonadal men was associated with increased flow-mediated dilation, a widely accepted surrogate marker of endothelial dysfunction. In contrast, chronic testosterone therapy decreased flow-mediated dilation in hypogonadal men, although statistical significance was not achieved for both effects, in part due to high heterogeneity [46]. DHT administration in male rats leads to hypertension by causing endothelial dysfunction [47,48].

In the current study, we used DHT to avoid confounding estrogenic effects of testosterone via conversion of testosterone to estrogen by aromatase [45]. Using molecular and functional assays, our in vitro studies demonstrate that the presence of androgen DHT exacerbated S1-induced-endothelial activation, as evidenced by increased transcript expression of cell adhesion molecules and the anti-fibrinolytic marker PAI-1, showing a cooperative effect of DHT in promoting S1-induced endothelial injury. In the current study, we relied on mRNA transcript expression of endothelial cell injury markers, and note that RNA structure is important for determining the functionality of expressed RNA and protein translation [49]. Modeling of RNA structure prediction may improve the molecular understanding and mechanisms described in the current study. The functional significance of the pro-inflammatory effect was validated by the finding of increased adhesion of THP-1 monocytes to the endothelial monolayer. Adhesion molecules such as E-selectin, VCAM1 and ICAM1 are known to promote monocyte adhesion via integrin interaction [50], whereas an elevated PAI-1 level promotes thrombosis by inhibiting tPA, all of which may contribute to pro-inflammatory and pro-thrombotic conditions in patients with COVID-19. Prior data in COVID-19 have shown that accumulation of CD68+ macrophages and activated cytotoxic CD8+ T cells are positively associated with diffuse alveolar damage [51]. Additionally, hyperplasia of type II pneumocytes and lung endothelium have been described [52,53], providing some further evidence of the determinantal effect of monocytes/macrophage infiltration and endothelium activation in COVID-19.

TMPRSS2 belongs to a family of serine proteases, and its role in cleaving and activating spike protein is well documented. TMPRSS2 is widely expressed in the lungs, nasal epithelium and gastrointestinal tract, and its regulation by androgens has been shown in the context of prostate cancer [54]. TMPRSS2 and ACE2 co-exist in several cells, including endothelial cells, pulmonary pneumocytic type II cells, human nasal cells, bronchial transient secretory cells, human and mouse conjunctiva, with increased expression in men and diabetics [55,56,57,58]. In the current study, we observed increased ACE2 and TMPRSS2 transcript expression following exposure to DHT. Increased TMPRSS2 transcript expression after DHT exposure in ECs was markedly higher compared to ACE2, which is consistent with reports linking TMPRSS2 as an androgen-regulated gene [54]. These data indicate a possible link between androgen-mediated ACE2 and TMPRSS2 regulation in COVID-19. This finding is concordant with data from androgen-mediated increases in ACE2 expression in human embryonic stem cell-derived cardiac cells and human primary alveolar epithelial cells and androgen-mediated regulation of TMPRSS2 in prostate cancer [59,60], supporting a hypothesis that higher ACE2 and TMPRSS2 expression are related to poorer prognosis in men infected with COVID-19. ARBs have been noted to increase ACE2 expression in animal models, and were therefore initially speculated to increase the risk of SARS-CoV-2 infection [26,27]. Clinical studies have consistently documented a lack of risk according to ARB treatment [27,61]. Our in vitro data are in line with these clinical findings, as we did not find any further increase in endothelial injury by SARS-CoV-2 spike protein in the presence of the ARB valsartan, despite the observation of increased endothelial ACE2 expression with ARB treatment in vitro.

Elevated circulating blood levels of the inflammatory cytokine, TNF-α have been described in the cytokine milieu of patients with cardiovascular disease and in those with COVID-19 [21,62]. TNF-α and other inflammatory cytokines can directly damage the homeostatic vascular endothelium by promoting immune cell adhesion, increased vascular permeability and capillary leak, which results in vascular and pulmonary alveolar dysfunction [63]. Our data provide evidence that in the presence of TNF-α, S1 enhanced expression of the endothelial cell adhesion markers in vitro and is consistent with the hypothesis that the presence of pro-inflammatory cytokines and chemokines increase the risk of poorer outcome with COVID-19. Additionally, increased anti-fibrinolytic PAI-1 and fibrinolytic t-PA by S1 in the presence of TNF-α is concordant with a recent report showing higher levels of PAI-1 and tPA that were associated with worse respiratory outcomes in COVID-19 patients [64], indicating complex fibrinolytic alterations in COVID-19.

The finding of sex differences in the levels of circulating endothelial injury markers in COVID-19 patients was validating of the in vitro findings; further studies will be needed to deeply explore inflammatory responses in humans. A prior study has reported increased levels of circulating E-selectin and VCAM-1 in ICU, as compared to non-ICU COVID-19 patients [19]. However, sex-difference in endothelial injury markers have not been reported. Here, we found significantly higher levels of VCAM-1 and E-Selectin in men as compared to women with COVID-19, suggesting clinically relevant exaggerated endotheliopathy in men, providing a potential mechanistic target associated with higher risk of critical illness and death after COVID-19 infection.

Finally, we tested the therapeutic potential of the FDA approved drug spironolactone, a commonly prescribed mineralocorticoid receptor antagonist that is safe and well-tolerated [65,66], which may serve as potential candidate for drug repurposing as a COVID-19 treatment. Spironolactone was introduced in 1959, and thus has a long safety record, with indications for use in the treatment of hypertension and heart failure, acting as potassium-sparing diuretic by blocking mineralocorticoid receptor activity [67]. Spironolactone is also prescribed to women to treat hyperandrogenic conditions due to its anti-androgenic activity [68,69]. Here, we showed that in the presence of DHT, spironolactone at levels corresponding to typically prescribed dosing, dramatically reduced S1-mediated transcript expression of cell adhesion and pro-thrombotic markers. Transcript-level effects were validated by demonstrating reduced THP-1 cellular adhesion to human aortic ECs, consistent with decreased vascular inflammation. Additionally, spironolactone reduced ACE2 transcript expression in ECs, suggesting that some of its beneficial effects may be in part mediated by lowering endothelial ACE2 expression. In the presence of valsartan, the benefit of spironolactone in S1-induced EC injury persisted, suggesting ARB use may not limit a potential beneficial effect of spironolactone. These data support the use of spironolactone in ameliorating inflammatory and thrombotic response in the treatment of COVID-19, which would need to be confirmed by a clinical trial.

It has been hypothesized that the beneficial effects of spironolactone may be mediated by reducing the binding of the S1 protein to the ACE2 receptor by downregulating furin and plasmin expression via nexin 1 or serine E2 (PN1) [23]. Furin and plasmin are believed to mediate additional proteolytic processing of S protein, and thus promote SARS-CoV-2 entry into the cells [23]. Further studies are needed to identify the role of PN1 in regulating furin- and plasmin-mediated ACE2 expression changes with spironolactone therapy.

Taken together, these data support a rationale for further testing of mineralocorticoid antagonism to attenuate tissue damage in COVID-19 mediated by endothelial injury, particularly in men. Early randomized clinical trials with a small sample size showed beneficial effects of anti-androgen therapy in mild-to-moderate and severe COVID-19 infection by improving the virus clearance, and reducing time to clinical remission [70,71,72]. Well-powered clinical trials are next needed to investigate a potential beneficial effect of mineralocorticoid antagonism to reduce the risk of COVID-19 adverse outcomes such as organ dysfunction, hospitalization and mortality. While generally well-tolerated, the safety of spironolactone with regards to potential hyperkalemia or worsening renal function would need to be considered in clinical studies [73].

## 5. Conclusions

In conclusion, our results provide new insights into the molecular mechanisms contributing to SARS-CoV-2 spike protein S1-mediated endothelial injury and evidence for a critical role of androgen and TNF-α signaling. Accordingly, these findings suggest relevant mechanisms in COVID-19-related disease severity in men and cardiovascular disease patients, and support a potential therapeutic role of spironolactone in the treatment of COVID-19.

## Figures and Tables

**Table 1:** Clinical characteristics of patients with COVID-19 of Cohort #1 (soluble VCAM-1) No. = number, BMI = Body mass index.

Clinical Characteristic	No. (n = 11)
Age, years	
Age < 40	6 (54.5%)
Age ≥ 40	5 (45.4%)
Men	8 (72.7%)
Requiring mechanical ventilation	8 (72.7%)
Body mass index, kg/m^2^	
BMI ≤ 30	1 (9%)
BMI 31–35	6 (54.5%)
BMI > 35	4 (36.3%)

**Table 2:** Clinical characteristics of patients with COVID-19 of Cohort #2 (soluble E-Selectin) No. = number, BMI = Body mass index.

Clinical Characteristic	No. (n = 242)
Age, years	
Age < 40	36 (14.9%)
Age ≥ 40	206 (85.1%)
Men	136 (56.2%)
Requiring mechanical ventilation	105 (43.38%)
Hypertension	136 (56.2%)
Diabetes	97 (40.08%)
History of smoking	66 (27.27%)
Body mass index, kg/m^2^	
BMI ≤ 30	111 (47.2%)
BMI 31–35	53 (22.6%)
BMI > 35	71 (30.2%)

## Data Availability

The data presented in this study are available on request from the corresponding authors.

## Supplementary Materials

The following are available online at https://www.mdpi.com/article/10.3390/v13112209/s1, Figure S1: In ECs, TNF-α significantly downregulated VWF expression in vitro, although co-administration of S1 and TNF-α did not further change VWF expression; Supplemental Table S1: Human qPCR primers.
