# Supplementary material for: SARS-CoV-2 Spike Protein S1-Mediated Endothelial Injury and Pro-Inflammatory State Is Amplified by Dihydrotestosterone and Prevented by Mineralocorticoid Antagonism
Source: Viruses. 2021 Nov 3;13(11):2209. doi: 10.3390/v13112209 (PMC8617813; doi:10.3390/v13112209)
Supplement: Supplementary file 1 [file viruses-13-02209-s001.zip › viruses-1415024-supplementary.pdf]

SUPPLEMENT INFORMATION

SARS-CoV-2 spike protein S1-mediated endothelial injury and pro-inflammatory state is amplified by dihydrotestosterone and prevented by mineralocorticoid antagonism

Nitin Kumar [1,2], Yu Zuo [3], Srilakshmi Yalavarthi [3], Kristina L. Hunker [1,2], Jason S. Knight [3], Yogendra Kanthi [1,4], Andrea T. Obi [5], and Santhi K. Ganesh [1,2*]

1 Department of Internal Medicine, Division of Cardiovascular Medicine, University of Michigan, Ann Arbor, MI, USA
2 Department of Human Genetics, University of Michigan, Ann Arbor, MI, USA
3 Division of Rheumatology, Department of Internal Medicine, University of Michigan, Ann Arbor, MI, USA
4 Laboratory of Vascular Thrombosis & Inflammation, National Heart, Lung and Blood Institute, National Institutes of Health, Bethesda, MD, USA
5 Section of Vascular Surgery, Department of Surgery, University of Michigan, Ann Arbor, MI, USA
* Correspondence: Santhi K. Ganesh, MD. 1150 West Medical Center Drive, MSRB 3 Room 7220A, Ann Arbor, Michigan 48109-0644, USA. Tel: (734)764-4500; Fax: (734)936-8266; Email: sganesh@umich.edu

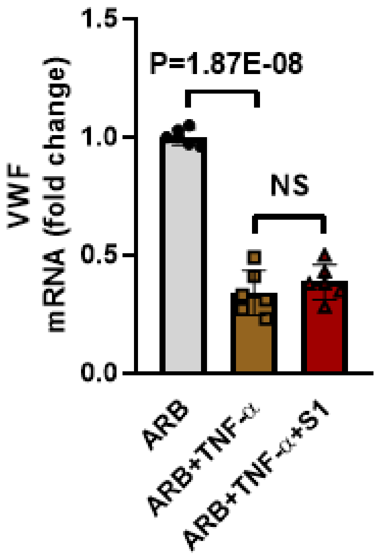

Figure S1: In ECs, TNF-α significantly downregulated VWF expression *in vitro*, however co-administration of S1 and TNF-α did not further change VWF expression.

Supplemental Table S1: Human qPCR primers.

Human Gene	Gene description	Forward Primer (5' > 3')	Reverse Primer (5' > 3')
SELE	selectin E	GTTCCTTCCTGCCAAGTGGT	GATCTTTCCCGGAACTGCCA
VCAM1	vascular cell adhesion molecule 1	CGAACCCAAACAAAGGCAGAG	CTCTGGGGGCAACATTGACA
ICAM1	intercellular adhesion molecule 1	GGAGCTTCGTGTCCTGTATGG	CTGGCACATTGGAGTCTGCT
SERPINE1	plasminogen activator inhibitor 1	CCTCAGGAAGCCCCTAGAGA	TGGAGAGGCTCTTGGTCTGAA
PLAT	plasminogen activator, tissue type	AGTGTGAGCTCTCCGGCTA	TCCGAATAGAAAGGAGACAAGGC
VWF	von Willebrand factor	GGAGATGTTTGCCTACGGCT	GAGCGTCTCATCACGCTTCA
RPL37	ribosomal protein L37	CGCAGATTCAGGCATGGAT	AGCTGCCCTCTTGGGTTTAGG
